# Emerging role of SETD2 in the development and function of immune cells

**DOI:** 10.1016/j.gendis.2025.101622

**Published:** 2025-04-03

**Authors:** Longmin Chen, Yuan Zou, Yan Dong, Tian Hong, Qianqian Xu, Jing Zhang

**Affiliations:** a Department of Respiratory and Critical Care Medicine, NHC Key Laboratory of Respiratory Disease, Tongji Hospital, Tongji Medical College, Huazhong University of Science and Technology, Wuhan, Hubei 430100, China; b Department of Rheumatology and Immunology, The Central Hospital of Wuhan, Tongji Medical College, Huazhong University of Science and Technology, Wuhan, Hubei 430014, China; c Department of Rehabilitation, Tongji Hospital, Tongji Medical College, Huazhong University of Science and Technology, Wuhan, Hubei 430000, China; d Department of Gerontology, The Central Hospital of Wuhan, Tongji Medical College, Huazhong University of Science and Technology, Wuhan, Hubei 430014, China

**Keywords:** Chromatin biological processes, Epidrug discovery, H3K36me3, Immune cells, SETD2

## Abstract

SET domain-containing 2 (SETD2) is a methyltransferase that catalyzes trimethylation of lysine 36 on H3 (H3K36me3) in mammals, an epigenetic mark associated with actively transcribed regions. SETD2 is implicated in multiple chromatin biological processes, such as alternative splicing, transcriptional regulation, DNA damage repair, and maintenance of genomic integrity. Extensive studies have demonstrated that *SETD2*-inactivating mutations and resultant dysregulation of these functions may result in tumorigenesis. However, the role of SETD2 in the development and function of immune cells receives relatively limited attention. In this review, we seek to summarize current knowledge of the biological function and underlying mechanisms of SETD2 and highlight its important role in immune cell biology. By influencing the biological processes of immune cells, SETD2 participates in the pathogenesis of immune-related diseases, including infection, cancers, autoimmune diseases, and inflammatory diseases. Finally, we discuss challenges and prospects for targeting SETD2 in immune cells to provide guidance for treating those diseases in clinical practice.

## Introduction

The genetic information of eukaryotic cells is stored in the chromatin, which is a DNA and histone protein macromolecular complex that facilitates the packaging of the genome into the nucleus.^1^^,^^2^ As the fundamental unit of chromatin, the nucleosome is composed of a ∼147-bp stretch of DNA that winds around an octamer of histones H2A, H2B, H3, and H4. Besides the action of histone variants that can be substituted for canonical histones and specialized protein machinery capable of assembling, disassembling, and moving nucleosomes along DNA, histone post-translational modifications exert important role in the regulation of chromatin-based processes,^3^ such as transcription, recombination, DNA repair, replication, and genome topology.^4^ The histone post-translational modification landscape is established and reset by antagonizing enzymes of “writers” and “erasers”, respectively, and proteins that recognize particular post-translational modifications via specific domains are referred to as “readers”.^1^^,^^4^ Histone post-translational modifications occur not only in the N-terminal histone protein tails protruding from the nucleosome but also on the lateral surface of the histone octamer that is in direct contact with DNA.^5^ Adding to this complexity, many of the histone-modifying enzymes utilize metabolites as substrates or cofactors, intimately linking their catalytic activity with cellular metabolic status.^6^^,^^7^ In recent years, an ever-growing list of histone post-translational modifications has been identified, including methylation, acetylation, ubiquitination, phosphorylation, benzoylation, succinylation, crotonylation, s-palmitoylation, and serotonylation.^4^

The effects of histone methylation are subtle and site-specific, as it can exist in three distinct states on both lysine (Kme1, Kme2, and Kme3) and arginine (Rme1, Rme2 symmetrical, and Rme2 asymmetrical) residues.^8^ The trimethylation of lysine 36 on H3 (H3K36me3) is enriched in actively transcribed genome regions.^9^^,^^10^ The fidelity of H3K36me3 deposition is essential to maintain genomic stability, and disturbance of the state has been closely linked to human diseases, such as prenatal developmental disorder,^11^^,^^12^ tumorigenesis,^13^, ^14^, ^15^ and immune-related diseases.^16^^,^^17^ These conditions are probably attributed to the defects in processes related to transcription elongation,^18^ alternative splicing,^15^ polycomb antagonism,^19^^,^^20^ DNA damage repair,^21^^,^^22^ and cell-cycle control.^23^ There are six major classes of histone lysine methyltransferase (KMT1-6) that transfer methyl groups to lysine residues, among which the KMT3 family primarily methylates H3K36. In mammalian cells, a number of enzymes can deposit the monomethylation and dimethylation marks on H3K36, while SET domain-containing 2 (SETD2, also known as KMT3A or HYPB) is the only known enzyme that catalyzes the trimethylation of H3K36.^15^ SETD2 was first found to be expressed in human cluster of differentiation (CD)34^+^ hematopoietic stem/progenitor cells in 1998.^24^ Soon after, it was identified as a huntingtin interacting protein that may participate in Huntington's disease pathogenesis, and was subsequently shown to possess a unique role in orchestrating histone code.^25^

Beyond H3K36me3-mediated regulation of gene transcription and DNA-based processes, SETD2 was also reported to have the capacity to modulate cellular signaling and responses via modification of non-histone substrates.^17^ Through both H3K36me3 and non-histone methylation, SETD2 participates in diverse physiological and pathological processes. In recent years, studies have revealed a role for SETD2 in immune system development,^26^^,^^27^ immune cell fate decision,^16^^,^^28^ pathogen recognition,^29^ and immune and inflammatory responses.^17^^,^^30^ Notably, an in-depth understanding of the correlation between SETD2 and immune cell biology will help us to elucidate the mechanisms underlying the development of immune-related diseases. Therefore, in this review, we provide an overview of the structure and biological function of SETD2, with a particular focus on its contribution to different types of immune cells. We will also summarize the implication of SETD2 dysregulation in immune disorders and the progress of several epigenetic drugs that target SETD2.

## The protein structure of SETD2

**Figure 1:**
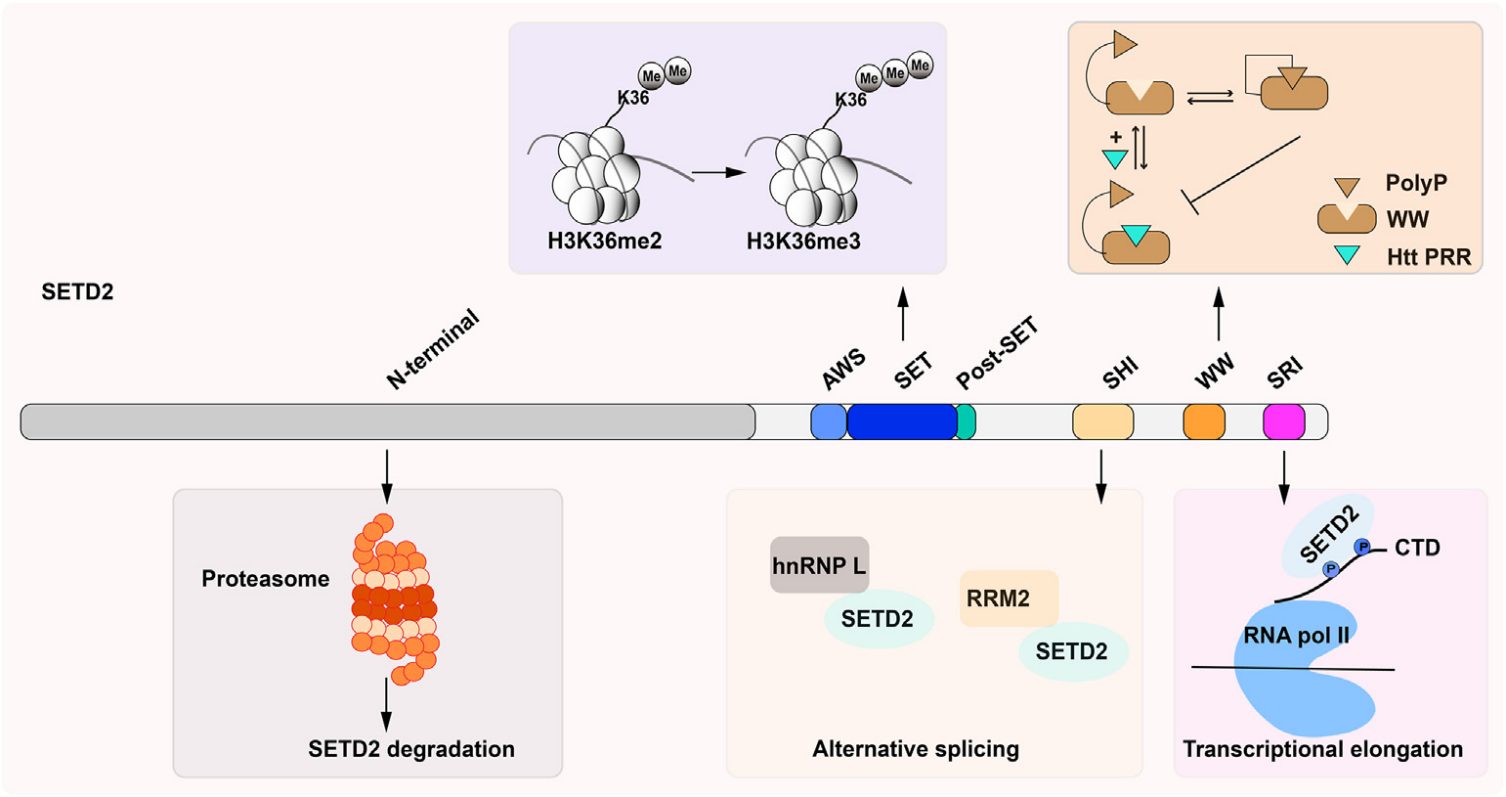
Protein structure of mammalian SETD2. SETD2, SET domain-containing 2; AWS, associated with SET domain; SHI, SETD2-hnRNP interaction domain; SRI, Set2-Rpb1 interacting domain; WW, tryptophan–tryptophan domain.

The mammalian SETD2 protein comprises several conserved functional domains, including the AWS (associated with SET)-SET-PostSET domains, a WW (tryptophan–tryptophan) domain, an SRI (Set2-Rpb1 interacting) domain, an SHI (SETD2-hnRNP interaction) domain, and a large unstructured N-terminal domain (Fig. 1).^31^ The SET domain lies between the AWS and post-SET domain and catalyzes the methylation of H3K36. The WW and SRI domains are protein-binding domains. The WW domain, named for two conserved tryptophan residues, generally interacts with proline-rich peptides. For example, the WW domain binds to a proline-rich region (PRR) in huntingtin (HTT), and this interaction is finely modulated intramolecularly by the polyproline stretch at the C-terminus of SETD2.^32^ It was recently reported that via its interaction with HTT and the actin-binding adapter HIP1R (HTT-interacting protein 1-related protein), SETD2 trimethylated lysine-68 (ActK68me3) in cells, which promoted actin polymerization and cell migration.^33^ The SRI domain mediates the binding of SETD2 to phosphorylated Ser2 and Ser5 on the C-terminal domain of RBP1, the largest subunit of RNA polymerase II (RNA Pol II).^34^^,^^35^ This allows SETD2 to be tethered to RNA Pol II, move along with the polymerase, and to methylate H3K36 in actively transcribed gene bodies during transcription. SETD2 associates with heterogeneous ribonucleoprotein L (hnRNP L), RNA recognition motif 2 (RRM2), and other mRNA processing factors through its SHI domain, which regulates a subset of alternative splicing events.^36^^,^^37^ Moreover, the deletion of the SHI domain results in a reduced deposition of H3K36me3, although the decrease is not as severe as that observed upon SRI deletion, indicating that the SHI domain is important for SETD2's methyltransferase activity.^36^ The N-terminal of SETD2 contains more than half of the protein sequence and contributes to the robust degradation of SETD2 through the ubiquitin-proteasome pathway.^38^

## Biological function and the underlying mechanisms of SETD2

**Figure 2:**
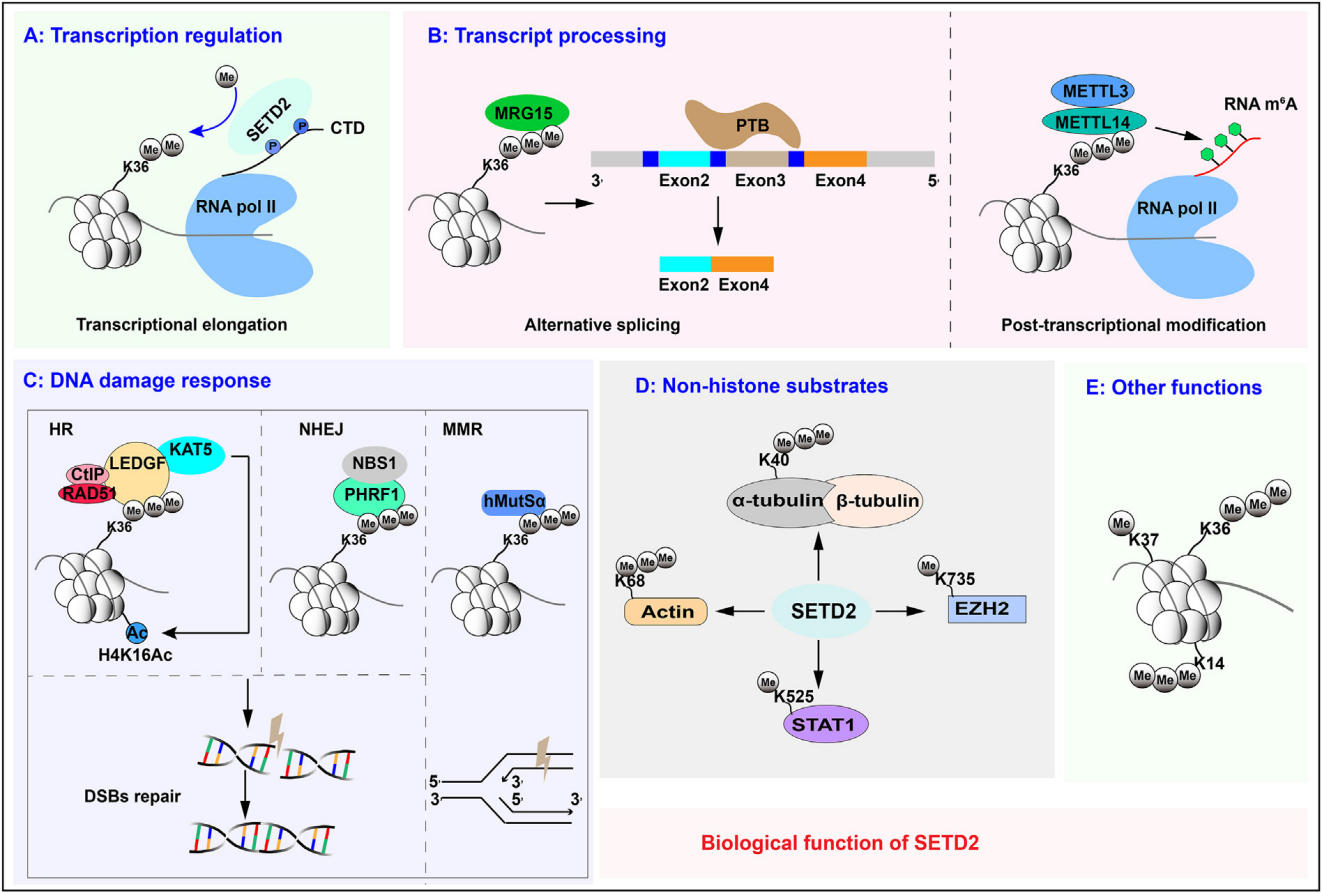
The schematic diagram of the biological function of SETD2. SETD2 participates in both physiological and pathological processes by modulating DNA damage response, alternative splicing, histone modifications, transcriptional elongation, m^6^A modification, and protein post-translational modification. SETD2, SET domain-containing 2.

Since its discovery over two decades ago, numerous studies have been dedicated to SETD2. Currently, the biological function of SETD2 and the molecular mechanisms underlying its role are well studied (Fig. 2), which will be discussed in this section.

### Transcription regulation

Transcription is carried out by RNA Pol II together with a large number of accessory proteins, which is a highly regulated and inherently stochastic process.^39^ After initiation, RNA Pol II enters the stage of transcription elongation. Elongating RNA Pol II interacts with the SRI domain in SETD2 via its phosphorylated C-terminal domain. SETD2 then piggy-backs on RNA Pol II to deposit H3K36me3 in the wake of RNA Pol II, especially along the 3′ end of the coding regions of actively transcribed genes. Although H3K36me3 is widely described to be associated with active chromatin, it has also been involved in transcriptional repression.^40^^,^^41^ This indicated that the transcriptional outcomes of H3K36me3 depend on chromosomal context, possibly in concert with other chromatin factors and modifications. Furthermore, SETD2-dependent FACT (facilitates chromatin transcription) recruitment is critical to reassemble nucleosomes during transcriptional elongation, thereby inhibiting cryptic intragenic transcription initiation.^18^ Intragenic transcription initiation has the potential to generate transcriptome diversity on one hand and might potentiate diseases such as cancer on the other. Consistent with this, *SETD2* mutations were identified in a large variety of human tumors,^42^, ^43^, ^44^ suggesting that further studies are required to elucidate its role in repressing intragenic transcription initiation in the context of cancer.

### Transcript processing

SETD2 engages in the processing of transcripts, such as alternative splicing and post-transcriptional modification. In multicellular eukaryotes, alternative splicing is one of the main sources of protein diversity.^45^ Genome-wide RNA profiling analyses have revealed a crucial role of SETD2 in splicing.^46^^,^^47^ The loss of SETD2-mediated H3K36me3 leads to widespread RNA processing defects, including exon skipping, intron retention, and alternative transcription start and termination sites, affecting nearly 25% of all expressed genes.^47^ H3K36me3 can regulate pre-mRNA splicing through various mechanisms. Strikingly, the MORF-related gene on chromosome 15 (MRG15) can bind to H3K36me3 and recruit splicing regulator polypyrimidine tract-binding protein (PTB) to its target alternatively spliced exons.^48^ In addition to MRG15, another chromatin-associated protein, PC4 and SF2 interacting protein 1 (Psip1), is also shown to recognize H3K36me3.^49^ The *Psip1* gene encodes splice variants p52 and p75, and the N-terminal Pro-Trp-Trp-Pro (PWWP) domain that binds to chromatin is common to both Psip1 isoforms. The PWWP domain of Psip1 p52 isoform binds to H3K36me3 and splicing factor Srsf1, contributing to the modulation of alternative splicing. Psip1/p75, however, is not implicated in splicing but interacts with H3K36me3 to enhance DNA repair.^50^ Furthermore, SETD2 not only regulates splicing, but splicing, in turn, influences the establishment of the normal pattern of H3K36me3. Inhibition of splicing impairs recruitment of SETD2 and reduces H3K36me3 in intron-containing genes, whereas splicing activation has the opposite effect.^51^^,^^52^

*N*^6^-methyladenosine (m^6^A) modification is the most common and abundant modification of mRNA, which plays a crucial role in diverse cellular processes by regulating mRNA biology.^53^ It has been reported that H3K36me3 is a general determinant for m^6^A deposition.^54^ H3K36me3 shows a similar coding sequence and 3′ untranslated region distribution pattern to m^6^A, and ∼70% of m^6^A peaks overlap with H3K36me3 sites.^53^ Mechanistically, methyltransferase-like 14 (METTL14), an essential m^6^A catalytic partner for METTL3 in the m^6^A methyltransferase complex, directly recognizes and binds to H3K36me3, which guides the m^6^A methyltransferase complex to actively transcribed nascent RNAs to deposit m^6^A co-transcriptionally.^54^ Therefore, m^6^A RNA modification serves as another mechanism of gene expression control for SETD2/H3K36me3, and the association between m^6^A and other histone modifications is expected to be uncovered in the near future.

### DNA damage response

Cells are constantly challenged by both endogenous and exogenous DNA damaging agents. If left unrepaired or misrepaired, these DNA lesions may cause the accumulation of DNA errors and genomic instability, which is associated with aging, cancer, neurodegeneration, and impaired immune system function. Similar to other DNA transaction-related processes, DNA damage response is extensively controlled by histone post-translational modifications.

Homologous recombination (HR) and non-homologous end-joining (NHEJ) represent the two major DNA double-strand break (DSB) repair pathways in eukaryotic cells. The balance between HR and NHEJ is regulated by several important determinants, such as DNA end structure, the time factor, DNA end resection, and the chromatin context.^55^ Under physiological conditions, these control mechanisms operate to ensure that the DSB repair pathway choice is appropriate for the cellular context, including the local chromatin environment and cell cycle phase. In mammalian cells, SETD2 facilitates DNA repair predominantly through HR.^56^ As mentioned above, the reader protein Psip1/p75 is constitutively associated with SETD2-dependent H3K36me3 via its PWWP domain. Chromatin-bound Psip1/p75 recruits C–terminal binding protein interacting protein (CtIP) to DSBs, thereby promoting DNA end resection and HR during S and G2 phases of cell cycle.^22^^,^^50^ Chromatin immunoprecipitation-sequencing analyses further reveal that preexisting H3K36me3-dependent chromatin interaction with Psip1/p75 is required for the recruitment of RAD51, an essential HR protein, to AsiSI-induced DSBs within transcribed genes.^57^ Thus, DSBs that occur within transcribed genes favor repair through HR. In addition, H3K36me3-bound Psip1/p75 promotes chromatin localization of the histone acetyltransferase KAT5, which catalyzes H4K16 acetylation to create an open chromatin environment that is permissive for DSB repair.^58^ Disrupting H3K36me3 levels by depleting SETD2, overexpressing H3K36me3 demethylase KDM4A, or an H3.3K36M transgene all result in reduced recruitment of repair proteins and HR repair events, highlighting the importance of H3K36me3 in HR repair.^22^ Meanwhile, HR might not be the only approach by which SETD2 promotes DSB repair because it has been discovered that the H3K36me3 reader PHD and ring finger domains 1 (PHRF1) interacts with Nijmegen breakage syndrome 1 (NBS1) to modulate NHEJ and stabilize genomic integrity upon DNA damage insults.^59^ These findings suggest a potential role for SETD2/H3K36me3 in establishing the appropriate HR-NHEJ equilibrium. However, we consider that the known control mechanisms might be just the tip of the iceberg and need further exploration.

SETD2 has also been proven to be dedicated to DNA mismatch repair (MMR). H3K36me3 is necessary *in vivo* to recruit the mismatch recognition complex hMutSα onto chromatin to be replicated.^60^ Once mispairs are introduced during DNA replication, hMutSα can quickly identify the mismatch to initiate the MMR reaction. Consistent with this, cells deficient in *SETD2* display an MMR-deficient phenotype characterized by microsatellite instability and an increased mutation frequency.^60^

### Non-histone substrates

While most of the above-described functions of SETD2 are intimately linked to its catalytic activity on H3K36, recent advances in mass-spectrometry-based proteomics have firmly established that the scope of lysine methylation extends far beyond histone proteins.^61^ The mitotic microtubule α-tubulin is the first non-histone substrate discovered for SETD2.^62^ SETD2 methylates α-tubulin at lysine 40 (α-TubK40me3) during mitosis and cytokinesis, and loss of α-TubK40me3 by *SETD2* deletion leads to mitotic spindle and cytokinesis defects, micronuclei, and polyploidy.^62^ Thus, microtubule methylation is an alternative mechanism by which SETD2 maintains genomic stability besides its role in DNA damage repair. Actin is another cytoskeletal target methylated by SETD2. Methylation of actin at lysine 68 (ActK68me3) regulates actin polymerization/depolymerization dynamics and function of the actin cytoskeleton, consequently promoting cell migration.^33^ SETD2 further mediates mono-methylation of STAT1 on lysine 525, which reinforces interferon-induced STAT1 activation and antiviral immune response.^17^ In the context of prostate cancer, SETD2 directly methylates enhancer of zeste homolog 2 (EZH2) at K735 to facilitate its degradation. Since EZH2 enables cells to acquire invasive traits, SETD2-mediated EZH2-K735me1 is functionally and clinically important to restrict prostate cancer metastasis.^63^ With the rapid development of genome editing and proteomic technologies, it is foreseeable that more non-histone substrates will be discovered and that the function of SETD2 in health and disease will be further revealed at the molecular level.

### Other functions

Intriguingly, the biological function of SETD2 is more than that. Of note, SETD2-dependent H3K36me3 gets involved in extensive crosstalk with other chromatin marks. In mature oocytes, H3K36me3 directs the proper establishment of DNA methylation but is mutually exclusive with H3K4me3 and H3K27me3.^12^ In the absence of maternal SETD2, DNA methylation is lost in transcribing regions, whereas H3K4me3 and H3K27me3 occur ectopically in former H3K36me3 territories, leading to embryonic lethality after implantation.^12^ Previous studies have reported that H3K36me3 guides *de novo* DNA methylation through interacting with DNA methyltransferases Dnmt3a and Dnmt3b.^64^, ^65^, ^66^ Moreover, H3K36me3 inhibits polycomb repressive complex 2 (PRC2) activity, rendering transcriptionally active chromatin refractory to PRC2-mediated H3K27me3.^19^^,^^20^ Recently, SETD2 has been identified to catalyze H3K14 trimethylation *in vitro* and *in vivo*.^67^ In response to replication stress, SETD2-mediated H3K14me3 recruits the replication protein A (RPA) complex to chromatin and thus promotes ATR (Ataxia telangiectasia and Rad3 related) activation, which protects stalled replication forks and maintains genome stability.^67^ However, unlike H3K36me3, a well-defined histone mark related to and enriched in actively transcribed genome regions, the correlation between H3K14me3 and transcription remains largely unknown.^68^ SETD2 might also mono-methylate H3K37.^69^ H3K37me1 controls DNA replication origin firing, protecting the genome from spurious replication events in yeast, but whether this role is conserved in mammals is currently unclear.^69^

## Role of SETD2 in immune cells

The common progenitor cells in the bone marrow, termed hematopoietic stem cells (HSCs), are the original source of all immune cells. Using hematopoiesis-specific *Setd2* knockout mouse models, it has been demonstrated that Setd2 has a crucial role in maintaining the balance between HSC self-renewal and differentiation.^70^^,^^71^ *Setd2* deletion impairs HSC self-renewal and leads to malignant transformation, likely due to DNA replication stress and genome instability.^70^ Similarly, results from another study also suggest that *Setd2*-deficient HSCs show a loss of stem cell identity and an increase in differentiation toward progenitors, which leads to HSC exhaustion.^71^

**Figure 3:**
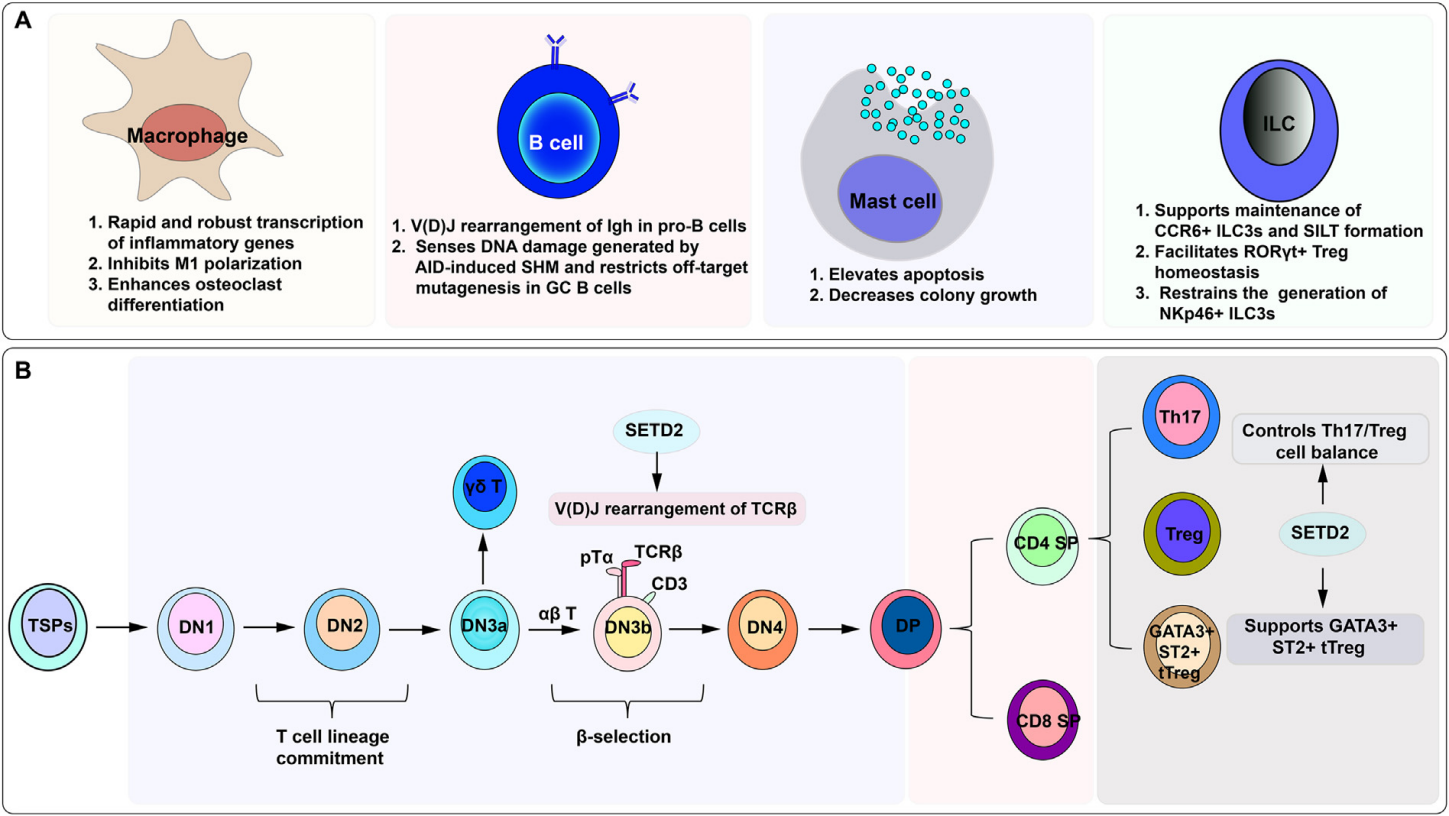
Summarized effects of SETD2 on different types of immune cells. SETD2 is essential for immune cell fate determination and function regulation. SETD2, SET domain-containing 2; AID, activation-induced cytidine deaminase; GC, germinal center; SHM, somatic hypermutation; TSPs, thymus seeding progenitors; DN, double negative; DP, double positive; SP, single positive.

Immune cells are divided into innate immune cells and adaptive immune cells based on their function and immune response patterns. The quick responding innate immune cells include monocytes/macrophages, dendritic cells, granulocytes, mast cells, and innate lymphoid cells (ILCs). Adaptive immune cells primarily contain T cells and B cells and have a delayed response that can take 4–5 days to fully develop but go on to form immunological memory.^72^ Based on accumulating evidence, SETD2 plays an important role in various biological processes of immune cells, including development, activation, polarization, and homeostasis, thereby regulating immune response and the development of diseases (Fig. 3).

### Macrophages

Macrophages are professional phagocytes that reside in specific anatomical locations throughout the body, enabling them to detect and respond to pathogens, metabolites, unfit cells, and environmental cues.^73^ Pathogen recognition by macrophages effectively engages conserved signaling pathways and transcription factors for rapid expression of inflammatory genes, such as *Tnf*, *Junb*, and *Nfkbia*.^74^^,^^75^ A growing body of evidence supports models in which dynamically regulated chromatin integrates with signal-activated transcription factors to play active roles in inflammatory gene induction. For example, owing to the constitutive activity of SETD2 during resting conditions, the elongation corepressor zinc finger MYND-type containing 11 (ZMYND11) is bound to pre-existing H3.3K36me3 in mouse bone marrow-derived macrophages. After stimulation with bacterial lipopolysaccharide, H3.3S31 phosphorylation introduces a steric clash that ejects ZMYND11 and enables SETD2 to bind, which in turn augments its enzymatic activity on H3.3K36 and triggers rapid and robust transcription.^30^ However, although stimulation-responsive genes are highly dependent on SETD2 for rapid, high-level expression *in vitro*, we can only predict that it might be the same situation *in vivo*.

Macrophages can adopt functionally distinct activation states depending on the context. Classically activated (M1) macrophages elicit inflammatory response to tissue damage and microbial pathogens by sensing damage-associated molecular patterns released from injured tissues and microbial components such as lipopolysaccharide.^76^ On the contrary, alternatively activated (M2) macrophages induced by interleukin (IL)-4 and IL-13 are effective at a resolution of inflammation and tissue repair. Epigenetic reprogramming and metabolic rewiring direct macrophage polarization and govern their functional plasticity,^77^^,^^78^ but how macrophages integrate these intricate regulatory circuits is not yet fully understood. Evidence suggests that SETD2 inhibits M1 macrophage polarization by suppressing hypoxia inducible factor 1 subunit alpha (HIF-1α)-mediated glycolysis, and this mechanism is controlled via SETD2-dependent H3K36me3 on hypoxia inducible factor 1 subunit alpha (*Hif1a*).^79^ As a result, the overexpression of *Setd2* attenuates sepsis-induced acute lung injury in mice.^79^ Down-regulation of SETD2 consistently results in elevated M1 macrophage polarization and glycolysis in acute suppurative osteomyelitis.^80^ In another study, less expression of SETD2 was observed in airway macrophages in patients with allergic asthma. SETD2 is necessary for the maintenance of IL-10 production in airway M2 macrophages, which contributes to immune homeostasis in the lung.^81^

Osteoclasts are a highly specialized macrophage population resident in the bone marrow.^82^ Osteoclasts are responsible for bone resorption, and their dysregulation is involved in all types of rheumatoid arthritis-associated bone erosion.^83^ Using gain- and loss-of-function approaches in the myeloid cell, Moonmoon et al confirmed that SETD2 increased Wnt5a expression and enhanced osteoclast differentiation.^84^ This discovery implies that SETD2 might serve as a potential target for the development of osteoclast-specific interventions to ameliorate bone loss in rheumatoid arthritis.

### Mast cells

Mastocytosis is a heterogeneous group of neoplasms characterized by the expansion and accumulation of neoplastic mast cells in one or more organ systems, including the skin, bone marrow, liver, spleen, lymph nodes, and gastrointestinal tract.^85^^,^^86^ Mastocytosis may present as cutaneous mastocytosis, systemic mastocytosis (SM), or local mast cell neoplasms, namely mastocytoma and mast cell sarcoma.^87^ According to histopathological and molecular features, organ involvement, and clinical variables, SM can be further subdivided into bone marrow mastocytosis, indolent SM, and smouldering SM (all considered non-advanced forms), and the advanced SM (AdvSM) entities: aggressive SM, mast cell leukemia, and SM with an associated hematologic neoplasm.^86^^,^^87^ A key driver of mast cell expansion and disease progression is the oncogenic machinery triggered by *KIT* mutations, most frequently the *KIT* D816V gain of function mutation.^86^ However, other lesions have also been detected in some forms of mastocytosis. It is worth noting that the loss of function of SETD2 is a recurrent event in AdvSM and mainly occurs at the post-translational level via the ubiquitin-proteasome pathway.^88^ Proteasome inhibitor rescues SETD2 protein expression and H3K36me3 level, leading to elevated apoptosis and decreased colony growth of HMC-1 cells and primary neoplastic mast cells from AdvSM patients.^88^ Results from a follow-up study showed that non-genomic loss of function of SETD2 in AdvSM was mediated by an Aurora kinase A (AURKA)/mouse double minute 2 (MDM2) axis. In this model, aberrant expression and activation of AURKA triggered MDM2-mediated ubiquitination of SETD2, which contributed to its proteasomal degradation and, consequently, to H3K36me3 deficiency.^89^ AURKA inhibitor alisertib was found to be at least as effective as KIT inhibitor in reducing mast cell colony growth and inducing apoptosis.^89^ However, additional mechanistic and clinical data are needed before SETD2 can be used for prognostication of SM patients and improvement of treatment approaches for AdvSM.

### Innate lymphoid cells

ILCs are a family of predominantly tissue-resident lymphocytes that do not express antigen-specific receptors but react to infection or insults through the generation of cytokines and secreted proteins.^90^^,^^91^ ILCs can be categorized into five subsets, including natural killer (NK) cells, T-bet^+^ ILC1s, GATA binding protein 3^+^ (GATA3^+^) ILC2s, retinoic acid receptor-related orphan receptor-γt^+^ (RORγt^+^) ILC3s, and lymphoid tissue inducer (LTi) cells.^90^^,^^92^ Among these, ILC3s are the most abundant subset in the intestine of both mice and humans, where they maintain intestinal homeostasis and promote the proliferation of intestinal stem cells. Intestinal ILC3s are composed of three key subpopulations: NKp46^+^ ILC3s, CCR6^−^NKp46^−^ double-negative ILC3s, and CCR6^+^ LTi-like ILC3s.^93^^,^^94^ Several landmark studies have defined the transcriptomes and cistromes of ILCs, including intestinal ILC3s.^95^, ^96^, ^97^ To comprehensively understand and delineate these regulatory programs, knowledge of epigenetic landscapes (*e.g.*, histone modifications, DNA modifications, and three-dimensional chromatin structure) is crucial. It has been shown that Setd2 determines genome accessibility and transcriptomic programs of ILC3s to regulate their fate commitment and intestinal immunity. Loss of *Setd2* in ILC3s causes increased generation of NKp46^+^ ILC3s with enhanced cytotoxic profile and anti-tumor capacity.^28^ The increased generation of NKp46^+^ ILC3 relies on RBP-Jκ, a pivotal nuclear mediator of the canonical Notch pathway. Meanwhile, *Rag1*^−/−^*Setd2*^*ΔILC3*^ mice manifest fewer LTi-like ILC3s and incompetent solitary intestinal lymphoid tissue formation, accompanied by decreased granulocyte-macrophage colony-stimulating factor (GM–CSF)–producing NKp46^−^ ILC3s and intestinal CD11b^+^CD103^+^ dendritic cells. The defective solitary intestinal lymphoid tissue formation and reduced GM-CSF production by NKp46^−^ ILC3s may collectively contribute to disturbed RORγt ^+^ Treg homeostasis and susceptibility to intestinal inflammation in *Rag1*^−/−^*Setd2*^*ΔILC3*^ mice upon T cell reconstitution.^28^

### T cells

T cells are the main cell components of the adaptive immune system, crucial for mediating cellular immune responses to maintain health and prevent diseases. T cells are developed from bone marrow-derived common lymphoid progenitor cells in the thymus and broadly grouped into CD4^+^ and CD8^+^ αβ T cells as well as rare populations of γδ T cells and NK T cells.^98^ Upon engaging with the thymic microenvironment, common lymphoid progenitor cells undergo multi-step developmental processes, including double-negative (DN) transition (DN1 to DN4), double-positive cell formation following positive selection, and the final single-positive stage after negative selection.^99^ At the DN3 stage, cells that rearrange genes encoding T cell receptor gamma (TCRγ) and TCRδ to generate a γδ TCR diverge from the major T cell lineage that expresses αβ TCR.^100^ Cells that rearrange the TCRβ coding locus are subject to a quality checkpoint called β-selection, which would make sure that T progenitors have rearranged a functional TCRβ gene before TCRα rearrangement. Descendants successfully completing β-selection and TCRα rearrangement up-regulate expression of the co-receptors CD4 and CD8 and differentiate into double-positive thymocytes.^101^

V(D)J recombination is initiated by the recombination activating 1/2 (RAG1/2) recombinase complex in a restricted lineage- and stage-specific manner. RAG binds to recombination signal sequences (RSSs) that flank each V, D, and J gene segment and causes DSBs.^102^ The DNA ends are then rejoined via the NHEJ repair pathway to generate a functional TCR gene.^103^ Therefore, V(D)J rearrangement is critically dependent on the DNA damage response and repair pathways. Given that H3K36me3 has been shown to be actively involved in DNA repair and SETD2 is the only known methyltransferase mediating H3K36me3 in mammals, *Setd2* conditional knockout mice are generated to investigate the role of Setd2 and Setd2-mediated H3K36me3 during T cell development. *Lck*-Cre^+^;*Setd2*^f/f^ mice display T cell lymphopenia due to the blockade of T cell development at the DN3 stage.^26^ Mechanistically, H3K36me3 catalyzed by Setd2 is significantly enriched in V/D/J segments located at or close to the RSSs of the TCRβ genes. The key recombinase Rag1 interacts with H3K36me3 in thymocytes, and *Setd2* deletion-mediated loss of H3K36me3 leads to decreased recruitment of Rag1 to the TCRβ gene loci and subsequent insufficient cleavage at RSSs. On the other hand, the expression of genes related to the DNA damage response and activation of ATM (Ataxia telangiectasia mutated), a major sensor of DSBs, are notably decreased in *Setd2* knockout lymphocytes. Therefore, suppressed occupancy of Rag1 on the TCRβ gene and the insufficient DNA damage response after the introduction of Rag1/2 complex-mediated DSBs at RSSs together contribute to the impaired V(D)J gene recombination in DN3 lymphocytes from *Lck*-Cre^+^;*Setd2*^f/f^ mice. Moreover, two missense *SETD2* mutations that impair the enzymatic activity of SETD2 are identified in primary immunodeficiency disorder patients, providing support for the clinical relevance of *SETD2* mutations in the development of human primary immunodeficiency.^26^ Interestingly, Setd2 ablation also favors the differentiation of the γδ T cell subset over the αβ T cell subset in mice,^26^ which is consistent with the results from another study.^104^ It is conceivable that γδ T cells have a smaller TCR repertoire and that V(D)J recombination does not occur as extensively in γδ T cells as in αβ T cells.

CD4^+^ T cells can be activated and differentiate into specialized effector subtypes, including Th1, Th2, Th9, Th17, Th22, Treg, follicular helper T (Tfh), and CD4^+^ cytotoxic T lymphocytes.^98^^,^^105^ Dysregulation of CD4^+^ T cell differentiation is closely related to various inflammatory and autoimmune diseases, such as inflammatory bowel disease, rheumatoid arthritis, and systemic lupus erythematosus.^106^ Therefore, dissecting the mechanisms that regulate CD4^+^ T cell differentiation into various subpopulations has important implications for a better understanding of disease pathogenesis and the development of innovative intervention strategies. Epigenetic modifications have been shown to cooperate with transcription factors to control the specificity and plasticity of T cell subsets by regulating chromatin structure and DNA accessibility. Recently, Setd2 was found to suppress Th17 but promote Treg differentiation through phospholipid remodeling.^16^ Setd2 up-regulates transcriptional expression of the phospholipid remodeling enzyme lysophosphatidylcholine acyltransferase 4 (Lpcat4) via directly catalyzing H3K36me3 to promote Lpcat4-mediated phosphatidylcholine PC(16:0,18:2) production. PC(16:0,18:2), in turn, inhibits endoplasmic reticulum stress and the transcriptional activity of HIF-1α, thereby controlling Th17/Treg cell balance. Consistent with this regulatory paradigm, CD4^+^ T cell-specific deletion of *Setd2* exacerbates neuroinflammation and demyelination in the experimental autoimmune encephalomyelitis model.^16^ Forkhead box P3 (Foxp3), the signature transcription factor of Tregs, is fundamental for Treg cell lineage determination and maintenance.^107^ Paradoxically, some activated Tregs express the transcription factors of effector CD4^+^ T cells, such as T-bet, GATA3, and RORγt, licensing Tregs to enter specific tissues or endowing them with a specialized function to restrain different Th cell responses.^108^, ^109^, ^110^, ^111^, ^112^ Notably, Treg expression of GATA3 plays a major role in tempering Th2 immunity in the skin and intestine.^113^^,^^114^ Suppression of tumorigenicity 2 (ST2) is the IL-33 receptor and is transcriptionally controlled by GATA3. GATA3^+^ Tregs are marked by the surface expression of ST2, which enhances the suppressive function of Tregs.^112^ There is evidence that Setd2 supports GATA3^+^ST2^+^ thymic-derived Tregs (tTregs) in the intestine by promoting the expression and reciprocal relationship of GATA3 and ST2.^115^ Consistent with the role of GATA3^+^ Tregs in suppressing Th2 cells, *Setd2* deficiency in Tregs leads to enhanced intestinal inflammation. Moreover, the authors observed elevated SETD2 expression in Tregs and reduced Th2 cells in cancerous tissues from colorectal cancer patients.^115^ The effect of Th2 cells in colorectal cancer is ambiguous; thus, elucidating the precise function of Th2 responses in colorectal cancer will facilitate the development and evaluation of novel cancer immunotherapeutics targeting SETD2 in Tregs.

Untreated HIV infection is typically associated with the progressive loss of CD4^+^ T cells and overactivation and functional exhaustion of CD8^+^ T cells, which will lead to opportunistic infections, malignancies, and ultimately, death.^116^^,^^117^ Whereas antiretroviral therapy is highly effective at eliminating free infectious virus, HIV persists in infected CD4^+^ T cells by entering a latent state.^118^ To achieve a globally available HIV cure, we need a greater understanding of the mechanisms that regulate reservoir maintenance and reactivation, especially from an epigenetic perspective. It is now recognized that H3K36me3 depletion by EPZ-719, a potent and selective SETD2 inhibitor, decreases post-integration viral gene expression and facilitates the emergence of latently infected cells.^29^ CRISPR/Cas9-directed knockout of *SETD2* in primary CD4^+^ T cells further confirmed the effect of SETD2 on HIV expression.^29^ Nevertheless, additional studies are required to determine the role of SETD2 in the control of the clinical reservoir.

### B cells

B cells are key components of the adaptive immune response and mediate humoral immunity.^119^ They can differentiate into either plasma cells or specific memory B cells to produce high-affinity antibodies and generate immunological memory. During early B cell development, RAG-dependent processes of V(D)J rearrangement of immunoglobulin (Ig) gene segments are now known to be able to generate an enormous amount of antibody diversity.^120^ Similar to TCRβ rearrangement, Setd2 is indispensable for immunoglobulin heavy chain (Igh) gene rearrangement. Ji et al report that V(D)J recombination of Igh is impaired in pro-B cells from *Cd19*-Cre^+^;*Setd2*^f/f^ mice, consistent with their observation that B cell development is arrested at the pro-B stage in *Mx1*-Cre^+^;*Setd2*^f/f^ mice.^26^ Results from the subsequent study suggest that loss of *Setd2*/H3K36me3 increases misrepair of RAG-induced DNA DSBs, particularly when combined with inactivation of the ATM kinase.^27^ Loss of *Setd2*/H3K36me3 additionally leads to a reduction in overall in B cell repertoire and a severe block in lymphogenesis.^27^

Germinal centers (GCs) are specialized microanatomical structures that form within the B cell follicles of secondary lymphoid organs upon exposure to antigen.^121^ A defining feature of the mature GC is its division into two major areas: a dark zone comprised almost exclusively of densely packed proliferating B cells referred to as centroblasts, and a light zone where smaller, non-dividing centrocytes interspersed within a mesh of stromal follicular dendritic cells, T cells, and macrophages.^122^ In GCs, B cells undergo rapid cell division and somatic hypermutation of the genes encoding immunoglobulin variable region (IgV) in the dark zone and affinity-based selection in the light zone.^123^ GC B cells migrate dynamically between these two compartments to experience iterative rounds of somatic hypermutation and selection, which is required for efficient affinity maturation. The somatic hypermutation process is associated with DNA strand breaks and requires activation-induced cytidine deaminase (AID). AID is an enzyme that initiates somatic hypermutation by deaminating cytidines directly on DNA, creating U:G mismatches that lead to point mutations in IgV genes upon repair.^124^ In GC B cells, heterozygous *Setd2* deficiency induces GC hyperplasia and dark zone polarization, which is mainly attributed to decreased apoptosis and increased completive fitness.^125^ Consistent with the fact that DNA damage generated by both AID-induced somatic hypermutation and via stress from rapid replication leads to GC B cell apoptosis, mechanistic studies revealed that the reduced apoptosis of *Setd2*^wt/−^ GC B cells was caused by a reduction in SETD2-dependent H3K36me3 and the resultant impaired DNA damage sensing. Moreover, *Setd2* haploinsufficiency-mediated H3K36me3 loss is associated with off-target AID mutations, predisposing to B-cell lymphomagenesis.^125^

Mutations in *SETD2* are frequently found in various types of lymphoid malignancies, including enteropathy-associated T-cell lymphoma,^126^ hepatosplenic T-cell lymphoma,^104^^,^^127^ diffuse large B-cell lymphoma (DLBCL),^125^^,^^128^ B-cell prolymphocytic leukemia,^129^ and B-cell progenitor acute lymphoblastic leukemia^130^ (for a comprehensive review see 131,132). Furthermore, SETD2 has been reported to interact with p53 and selectively regulate its downstream genes.^133^ The loss of *SETD2* inactivates p53 and results in the eviction of the G1/S checkpoint following DNA damage.^21^ In addition to direct protein–protein interaction, it is also possible that the defects in the DNA damage signaling in SETD2-deficient cells could extend to improper p53 activation. In recent years, evidence has emerged that by directly activating expression of genes involved in immunity, p53 has a broader role in homeostatic regulation of crucial aspects of immune responses, including pathogen sensing, antigen presentation, cytokine production, inflammasome formation and activation, NK cell cytotoxicity, T/B lymphocyte activation, *etc*..^134^^,^^135^ Although no relative experiment has been reported so far, it will be interesting to assess whether SETD2 has implications in immune cells through a mechanism involving p53 in the future.

## Advances in epidrugs targeting SETD2

Dysregulation of epigenetic processes is increasingly appreciated as a major contributing factor to multiple diseases. As a result, efforts in combating dysregulated epigenetic mechanisms contribute to the development and application of epidrugs, that is, drugs that target epigenetic modulators. Currently, the US FDA has approved three classes of epigenetic inhibitors, including DNA methyltransferase, histone deacetylase, and EZH2 inhibitors, and many others are undergoing clinical trials.^136^ With regard to H3K36me3, N-alkyl sinefungin analogues were identified as potent and selective SETD2 inhibitors by matching distinct transition-state characters of SETD2.^137^ However, because sinefungin is cell-impermeable, it cannot be utilized for cellular studies. Bajusz et al reported three non-nucleoside compounds with novel chemical scaffolds and experimentally confirmed SETD2 inhibitory activities. Among them, the component C13 effectively decreased H3K36me3 levels and inhibited the SETD2-dependent proliferation of two acute myeloid leukemia cell lines, prioritizing its chemotype as a viable starting point for optimized SETD2 inhibitors.^138^ In addition, docking and molecular dynamics simulation indicated that the traditional Chinese medicine compounds coniselin and coniferyl ferulate had high binding affinity and stable interactions with SETD2, which were potential lead compounds for future drug development.^139^ A screen of the epizyme proprietary histone methyltransferase-biased library identified two hits of interest, and structure-based drug design and drug metabolism/pharmacokinetics optimization led to EPZ-719, a novel SETD2 inhibitor with high potency and selectivity.^140^ Subsequently, EZM0414 was discovered as a selective, potent, and orally bioavailable SETD2 inhibitor by the same team.^141^ In both *in vitro* and *in vivo* preclinical studies, targeting SETD2 with EZM0414 significantly reduced the growth of t(4; 14) multiple myeloma (MM), as well as non-t(4; 14) MM and DLBCL cell lines.^142^ A phase I/Ib clinical trial has been initiated to evaluate safety and determine the optimal dose of EZM0414. After the dose-ranging phase, the study will be expanded to evaluate EZM0414 in t(4; 14) MM, non t(4; 14) MM, and DLBCL patient cohorts.

Despite promising reports on the development of small molecule inhibitors of SETD2, they are mainly confined to the treatment of certain hematological malignancies. However, whether these novel compounds have immunomodulatory effects remains largely unknown. Furthermore, there is a common concern regarding the unwanted adverse and off-target effects when treated with epidrugs.^143^ To minimize side effects, precise recognition of epidrug targets is required to develop selective enzyme inhibitors for specific cells. Assessment of the lowest effective dose and the use of an appropriate drug delivery system with desired release kinetics also help to overcome this issue.

## Concluding remarks

SETD2 is the only characterized methyltransferase responsible for the trimethylation of H3K36 and plays a critical role in multiple important cellular processes. SETD2 has been shown to affect gene/protein expression through various mechanisms involving the regulation of DNA methylation, DNA damage response, alternative splicing, histone modifications, transcriptional elongation, m^6^A modification, and protein post-translational modification. Early studies primarily focused on the function of SETD2 in tumors and nonimmune cells. In recent years, however, rapid progress has been made in understanding its role in immune cells. SETD2 can dictate immune cell fate and modulate immune cell functional behavior, thereby regulating the immune response and contributing to the pathogenesis of immune-related diseases. In the meantime, tremendous advances in high-throughput technologies facilitate the screening and discovery of epigenetic drugs targeting SETD2. Despite these overall gains, there remain some questions that should be taken into consideration. First, it will be necessary to elucidate the environmental stimuli and intracellular upstream signals that modulate SETD2 activity and H3K36me3 status. Second, the existing researches mainly concentrate on a few immune cells while overlooking other immune cells, so further exploration of the effect of SETD2 on various immune cells needs to be done. Third, targeting SETD2 for the treatment of immune-related diseases is still in the theoretical stage, and some SETD2 inhibitors have not been tested in clinical trials and lack safety assessments. Resolving these issues will be essential to fully understand the role of SETD2 in immune cell biology under physiological and pathological conditions and provide novel insights for clinical translation.

## CRediT authorship contribution statement

**Longmin Chen:** Writing – original draft, Funding acquisition. **Yuan Zou:** Data curation. **Yan Dong:** Data curation. **Tian Hong:** Visualization. **Qianqian Xu:** Visualization. **Jing Zhang:** Writing – review & editing, Supervision, Funding acquisition, Conceptualization.

## Funding

Our study was supported by the 10.13039/501100001809National Natural Science Foundation of China (No. 82300929, 82470877, 82100892), the 10.13039/501100018806Department of Science and Technology of Hubei Province Program Project (China) (No. 2022CFB739), the Intramural Research Program of the Central Hospital of Wuhan, Hubei, China (No. 23YJ14, 21YJ01), and Wuhan Talent Project (China).

## Conflict of interests

The authors declared no competing interests.
